# Risk factors and clinical outcomes of acute myeloid leukaemia with central nervous system involvement in adults

**DOI:** 10.1186/s12885-015-1376-9

**Published:** 2015-05-02

**Authors:** Chieh-Lung Cheng, Chi-Cheng Li, Hsin-An Hou, Wei-Quan Fang, Chin-Hao Chang, Chien-Ting Lin, Jih-Luh Tang, Wen-Chien Chou, Chien-Yuan Chen, Ming Yao, Shang-Yi Huang, Bor-Sheng Ko, Shang-Ju Wu, Woei Tsay, Hwei-Fang Tien

**Affiliations:** 1 Division of Hematology, Department of Internal Medicine, National Taiwan University Hospital, College of Medicine, National Taiwan University, No. 7, Chung-Shan South Road, Taipei, 100 Taiwan; 2 Taicheng stem cell therapy center, National Taiwan University, Taipei, Taiwan; 3 Taiwan Clinical Trial Bioinformatics and Statistical Center, Training Center, and Pharmacogenomics Laboratory, Taipei, Taiwan; 4 Department of Medical Research, National Taiwan University Hospital, College of Medicine, National Taiwan University, Taipei, Taiwan; 5 Department of Laboratory Medicine, National Taiwan University Hospital, College of Medicine, National Taiwan University, Taipei, Taiwan

**Keywords:** Adult, Acute myeloid leukaemia, Central nervous system, Prognosis, Risk factors

## Abstract

**Background:**

Acute myeloid leukaemia (AML) with central nervous system (CNS) involvement in adults is uncommon, and studies of this subject are scant.

**Methods:**

We conducted a retrospective study to investigate the clinical aspects, cytogenetic abnormalities, molecular gene mutations and outcomes of adult AML patients with CNS involvement. Three hundred and ninety-five patients with newly diagnosed AML were reviewed.

**Results:**

Twenty (5.1%) patients had CNS involvement, including 7 (1.8%) with initial CNS disease and 4 (1%) who suffered an isolated CNS relapse. The patients with CNS involvement were younger, had higher leukocyte, platelet, and peripheral blast cell counts, FAB M4 morphology, and chromosome translocations involving 11q23 (11q23 abnormalities) more frequently than did the patients without CNS involvement. No differences in sex, haemoglobin levels, serum LDH levels, immunophenotype of leukaemia cells, or molecular gene mutations were observed between the two groups. Multivariate analyses showed that age ≤ 45 years (OR, 5.933; 95% CI, 1.82 to 19.343), leukocyte counts ≥ 50,000/μl (OR, 3.136; 95% CI, 1.083 to 9.078), and the presence of 11q23 abnormalities (OR, 5.548; 95% CI, 1.208 to 25.489) were significant predictors of CNS involvement. Patients with initial CNS disease had 5-year overall survival and relapse-free survival rates that were similar to those without initial CNS disease. However, three of four patients who suffered an isolated CNS relapse died, and their prognosis was as poor as that of patients who suffered a bone marrow relapse.

**Conclusion:**

CNS involvement in adult patients with AML is rare. Three significant risk factors for CNS involvement including age ≤ 45 years, leukocyte counts ≥ 50,000/μl and the presence of 11q23 abnormalities were identified in this study. Future investigations to determine whether adult AML patients having these specific risk factors would benefit from CNS prophylactic therapy are necessary.

**Electronic supplementary material:**

The online version of this article (doi:10.1186/s12885-015-1376-9) contains supplementary material, which is available to authorized users.

## Background

Central nervous system (CNS) involvement in adults with acute myeloid leukaemia (AML) is uncommon, and its incidence is far less than that in patients with acute lymphoblastic leukaemia [1,2]. Most descriptions of the clinical features of AML with CNS involvement are from studies of paediatric AML patients [3-6]. The incidence of CNS involvement in childhood AML ranges from 6% to 29% [5-9]. Previous studies have shown that age < 2 years, high white blood cell (WBC) and peripheral blast cell (PBC) counts at diagnosis, French-American-British (FAB) M4 and M5 morphology, inversion of chromosome 16, and a hyperdiploid cytogenetic profile are risk factors for CNS involvement in paediatric AML patients at diagnosis [6-8]. On the other hand, isolated CNS relapse in paediatric patients with AML is associated with age < 2 years, high WBC count, hepatosplenomegaly, CNS involvement at diagnosis, FAB M5 morphology, and chromosome 11q abnormalities [10]. Reports of the outcomes of paediatric AML patients with CNS involvement have varied. Certain studies have shown that CNS involvement confers a poor prognosis, [11,12] whereas others have shown that it exerts no effect on survival [5,7,8,10].

The clinical features and treatment outcomes of adult AML patients with CNS involvement have not been well characterised. Peterson et al [13] described adult patients with acute nonlymphocytic leukaemia with CNS involvement, but their sample size was relatively small. Shihadeh et al [14] examined the cytogenetic profiles of AML patients with CNS disease in a cohort of 1354 patients in the United States, but did not examine the clinical outcomes. Rozovski et al [15] used the same cohort as Shihadeh et al and found that high serum levels of lactate dehydrogenase (LDH) at diagnosis, African-American ethnicity, and young age were risk factors for CNS involvement. Patients who achieved complete remission (CR) after induction chemotherapy had shorter disease-free survival and overall survival (OS) if they had CNS involvement. Bar et al [16] reported risk factors and outcomes for CNS AML involvement in patients at the pre-haematopoietic stem cell transplantation (HSCT) evaluation. Covariates associated with CNS involvement were higher WBC counts at diagnosis, prior CNS or other extramedullary disease, and disease status at pre-HSCT evaluation. Presence of CNS involvement at pre-HSCT evaluation had no impact on post-HSCT outcome. However, participants in their study were limited to those who had undergone HSCT, which may have confounded their findings. In this study, we investigated the clinical characteristics and outcomes of a large cohort of adult AML patients in Taiwan to determine the risk factors and outcomes associated with CNS involvement in AML. Several characteristics which had not been included in previous studies, such as the immunophenotype of leukaemia cells and 13 relevant molecular gene mutations in AML, were also examined. In addition, we investigated the impact of CNS involvement on outcomes including separate analyses on those with initial CNS disease and those with isolated CNS relapse.

## Methods

### Participants

Our study was approved by the Institutional Review Board of National Taiwan University Hospital (NTUH), and was performed in accordance with the Declaration of Helsinki. All participants provided signed, informed consent before participation in our study. Patients aged ≥ 18 years who were newly diagnosed with AML at NTUH between January 2000 and December 2008 were reviewed for enrollment. The diagnosis and classification of AML were made according to the criteria of the FAB Cooperative Group by the patient’s primary care haematologist, an expert in FAB classification. Patients with acute promyelocytic leukaemia (FAB M3 subtype), antecedent haematological diseases or therapy-related AML were excluded from our study.

A total of 395 AML patients were included in our study, amongst whom 280 (70.9%) received standard induction chemotherapy (idarubicin 12 mg/m^2^ per day for three days and cytarabine 100 mg/m^2^ per day for seven days) and then consolidation chemotherapy with three to four courses of high-dose cytarabine (2000 mg/m^2^ every 12 hours for four days, total eight doses), with or without an anthracycline (idarubicin or mitoxantrone), after achieving CR [17]. The remaining 115 patients received palliative therapy with supportive care and/or low-dose chemotherapy because of underlying comorbidity or the patient’s request. Allogeneic HSCT was performed in 97 patients. Our treatment protocols did not routinely include CSF tested at diagnosis or CNS prophylactic therapy, such as intrathecal chemotherapy. Lumbar puncture was performed only if clinically indicated. Intrathecal chemotherapy in combination with systemic chemotherapy, including high dose cytarabine, was adopted as the CNS-directed treatment for patients with CNS involvement. Patients who failed to respond completely to intrathecal or systemic chemotherapy, and those who had CNS granulocytic sarcoma and/or cranial nerve impairment, received radiation therapy. The median dose of radiation given to patients was 24 grays (Gy) (range, 20 to 25 Gy).

### Data collection

We retrospectively reviewed the clinical characteristics, cytogenetic profiles, molecular gene mutations and outcomes of patients in our AML cohort. A diagnosis of CNS involvement required the confirmation of leukaemic blast cells in the centrifuged cerebrospinal fluid (CSF) with the presence of more than five WBCs in the CSF [10,18] by at least two haematologists or the detection of a CNS granulocytic sarcoma using computed tomography or magnetic resonance imaging. Two clinical disease entities, initial CNS disease and isolated CNS relapse, were defined. Initial CNS disease consisted of the following conditions: (1) CNS involvement on the date of the initial AML diagnosis or (2) CNS involvement after receiving standard induction therapy and the absence of blast cells in the peripheral blood. Isolated CNS relapse was defined as a CNS relapse that was the first event following CR without evidence of bone marrow (BM) or other extramedullary relapse within 30 days [10]. The age, sex, FAB morphology subtype, haemogram, PBC, serum LDH level, immunophenotype of leukaemia cells, cytogenetics and molecular gene mutation at diagnosis of patients with CNS involvement were compared with those of patients without CNS involvement. Follow-up data were collected until death, loss to follow-up, or the end of the study period, June 30, 2011.

### Immunophenotyping

Monoclonal antibodies to the myeloid-associated antigens CD13, CD33, CD11b, CD15, and CD14, the lymphoid-associated antigens CD2, CD5, CD7, CD19, CD10, and CD20, and the lineage-nonspecific antigens HLA-DR, CD34, and CD56 were used to characterise the immunophenotype of the leukaemia cells as previously described [19].

### Cytogenetics

The BM cells were collected from AML patients for immediate cytogenetic analysis, or they were cultured for 1 to 3 days without exogenous stimulation before cytogenetic analysis as described previously [20]. The metaphase cells were banded using the trypsin-Giemsa technique, and karyotyped according to the International System for Human Cytogenetic Nomenclature (ISCN 2009) [21].

### Mutation analysis

Mutation analysis of 13 relevant molecular marker genes, including *NPM1*, [22] *CEBPA*, [23] *FLT3*-internal tandem duplication, [24] *RAS*, [25] *KIT*, [26] *ML*L-partial tandem duplication, [27] *WT1*, [28] *AML1/RUNX1*, [29] *ASXL1*, [30] *IDH1*, [31] *IDH2,* [32] *TET2,* [33] and *DNMT3A* [34] was performed as previously reported. Abnormal sequencing results were confirmed by at least two repeated analyses.

### Statistical analysis

The categorical data for patients with CNS involvement were compared with those of patients without CNS involvement, using a chi-squared analysis or the Fisher exact test. The Mann-Whitney *U* test was used to compare the medians of the continuous variables. The univariate and multivariate logistic regression analyses were used to identify factors predictive of CNS involvement. Variables that met a significance level of < 0.2 in the univariate analysis were included in the multivariate logistic regression analysis. The odds ratio (OR) from this analysis was used as a measure of the relative risk. OS was measured from the date of the first diagnosis to the end of the follow-up period, death from any cause, or the date of the last known follow-up examination. Relapse was defined as a reappearance of ≥ 5% leukaemic blast cells in a BM aspirate or newly developed extramedullary leukaemia in patients with a previously documented CR [35]. Relapse-free survival (RFS) was measured from the date of attaining a leukaemia-free state until the end of the follow-up period, the date of AML relapse, death from any cause, or the last known follow-up examination, whichever came first [35]. The Kaplan-Meier method was used to estimate the OS and the RFS, and the log-rank test was used to examine the significance of differences between the two groups. A two-sided *P* value < .05 was considered to indicate a statistically significant difference. The entire cohort was included the analyses of the correlation between CNS involvement and the clinical characteristics. All patients with CNS involvement received standard induction chemotherapy. Those in the palliative group received heterogeneous treatment strategies. Hence only the patients who received conventional standard chemotherapy were included in the analysis of survival. All statistical analyses were performed using the SPSS, Version 17, computer software (IBM, Armonk, NY, USA).

## Results

### Characteristics of the patients

Of the 395 AML patients included in our study, 20 (5.1%) developed CNS involvement, amongst whom seven (1.8%) had initial CNS disease and four (1%) suffered an isolated CNS relapse. Six patients (1.5%) suffered a CNS relapse concurrent with or subsequent to a marrow relapse, and three patients (0.8%) were diagnosed with primary refractory disease with subsequent CNS involvement. The clinical and laboratory data of patients with or without CNS involvement are shown in Table 1. Patients with CNS involvement had a lower median age (37.5 vs 54 y, *P* < .001), and exhibited higher WBC, PBC, and platelet counts at diagnosis than those of patients without CNS involvement. In addition, patients with CNS involvement exhibited FAB-M4-subtype morphology more frequently (*P =* .02) than did patients without CNS involvement. No differences in sex, haemoglobin levels, serum LDH levels, or the immunophenotype of leukaemia cells (Additional file 1: Table S1) were observed between the two groups.

**Table 1:** **Comparison of clinical and laboratory characteristics of patients with and without CNS involvement**

Variables	Total (n = 395)	Patients with CNS involvement (n = 20)	Patients without CNS involvement (n = 375)	*P*value
**Sex**†				0.648
Male	220	10(50)	210(56)	
Female	175	10(50)	165(44)	
**Age (year)**‡	53(18-90)	37.5(21-72)	54(18-90)	<0.001
**Laboratory data**‡				
WBC (/μL)	21950(120-423000)	83865(1860-277250)	21050(120-423000)	0.003
Haemoglobin (g/dL)	8.1(2.9-14.5)	8.1(4.9-14.5)	8.1(2.9-14)	0.392
Platelet (×1,000 /μL)	45(1-802)	71.5(11-255)	44(1-802)	0.03
PBC (/μL)	9014(0-369070)	42751(0-260615)	8360(0-369070)	0.009
LDH (U/L)	859(206-13130)	1641.5(265-8693)	842(206-13130)	0.069
**FAB**†				
M0	11	0(0)	11(2.9)	>0.999
M1	85	4(20)	81(21.6)	>0.999
M2	139	3(15)	136(36.3)	0.057
M4	116	11(55)	105(28)	0.02
M5	20	1(5)	19(5.1)	>0.999
M6	15	0(0)	15(4)	>0.999
Undetermined	9	1(5)	8(2.1)	0.377

†number of patients (%).

‡median (range).

Abbreviation: *CNS* central nervous system, *WBC* white blood cell, *PBC* peripheral blast cell, *LDH* lactate dehydrogenase, *FAB* French-American-British.

### Correlation of CNS involvement with cytogenetics and molecular gene mutations

Cytogenetic data were available for 378 patients at diagnosis. The karyotype characteristics of the AML patients are shown in Table 2. Patients with CNS involvement had a significantly higher incidence of the chromosome translocations involving 11q23 (11q23 abnormalities) (21.1% vs 2.8%, *P* = .003) than did those without CNS involvement. Data on molecular gene mutations of the AML patients are shown in Additional file 1: Table S2. The molecular gene mutations observed in the two groups did not differ.

**Table 2:** **Comparison of chromosomal abnormalities** ^*^ **seen in patients with and without CNS involvement**

Variables	Total (n = 378)	Patients with CNS involvement (n = 19)	Patients without CNS involvement (n = 359)	*P*value
		Number (%) of patients		
**Karyotype** ^†^				0.378
Favorable	50	3(15.8)	47(13.1)	
Intermediate	264	15(78.9)	249(69.4)	
Unfavorable	64	1(5.3)	63(17.5)	
t(8;21)	34	1(5.3)	33(9.2)	>0.999
inv(16)	16	2(10.5)	14(3.9)	0.189
11q23 abnormalities	14	4(21.1)	10(2.8)	0.003
-5/5q-^‡^	1	0(0)	1(0.3)	>0.999
-7/7q-^‡^	6	0(0)	6(1.7)	>0.999
t(7;11)	4	0(0)	4(1.1)	>0.999
t(6;9)	2	0(0)	2(0.6)	>0.999
+8^‡^	17	1(5.3)	16(4.5)	0.592

*Three hundred and seventy-eight patients had cytogenetic profiles at diagnosis. The remaining 17 patients did not have data of cytogenetic profiles because of inadequate metaphase cells for alalysis.

^†^Favorable, t(8;21), inv (16); unfavorable, -7, del(7q), -5, del(5q), 3q abnormality, complex abnormalities; Intermediate, normal karyotype and other abnormalities.

^‡^Includes only simple chromosomal abnormalities with 2 or fewer changes, but not those with complex abnormalities with 3 or more aberrations.

Abbreviation: *CNS* central nervous system.

### Risk factors associated with CNS involvement

In multivariate logistic regression analysis including variables significantly associated with CNS involvement in univariate analysis (Table 3), the independent risk factors were age ≤ 45 years (OR, 5.933; 95% CI, 1.82 to 19.343), WBC counts ≥ 50,000/μL (OR, 3.136; 95% CI, 1.083 to 9.078), and the presence of 11q23 abnormalities (OR, 5.548; 95% CI, 1.208 to 25.489).

**Table 3:** **Univariate and multivariate analyses to identify the risk factors predictive of CNS involvement**

Variable	Univariate analysis			Multivariate analysis		
	**OR**	**95% CI**	***P*** **value**	**OR**	**95% CI**	***P*** **value**
Age*	5.654	2.01-15.904	0.001	5.933	1.82-19.343	0.003
Sex	0.786	0.319-1.932	0.599	-	-	-
WBC†	4.069	1.616-10.244	0.003	3.136	1.083-9.078	0.035
Platelet§	2.303	0.755-7.026	0.143	2.804	0.799-9.837	0.107
LDH‡	1.931	0.688-5.425	0.212	-	-	-
FAB M4¶	3.143	1.266-7.803	0.014	2.023	0.646-6.341	0.227
inv(16) ^*∆*^	2.899	0.609-13.79	0.181	1.176	0.277-10.612	0.562
11q23 abnormalities^#^	9.307	2.615-33.123	0.001	5.548	1.208-25.489	0.028

Only variables with *P* value < 0.2 in the univariate analysis were incorporated into the multivariate logistic regression analysis.

*Age ≤ 45 y relative to age > 45 y.

†WBC greater than or equal to 50,000/μL vs less than 50,000/μL.

§Platelet greater than or equal to 30,000/μL vs less than 30,000/μL.

‡LDH greater than or equal to two times the upper limit of normal vs less than two times the upper limit of normal.

¶French-American-British M4 morphology vs others.

^*∆*^Inversion of chromosome 16 vs others.

^#^chromosome translocations involving 11q23 vs others.

Abbreviation: CNS, central nervous system; OR, odds ratio; CI, confidence interval; WBC, white blood cell; LDH, lactate dehydrogenase.

### Clinical characteristics and outcomes of patients with initial CNS disease

The clinical features and treatment outcomes of the patients with initial CNS disease (*n* = 7) are summarized in Table 4. Patients with initial CNS disease had higher WBC counts at diagnosis (*P* = .041) than those without initial CNS disease. Furthermore, the incidence of the chromosome 16 inversion was significantly higher (*P* = .001) in patients who had initial CNS disease than in patients without initial CNS disease.

**Table 4:** **Clinical characteristics and treatment outcomes of patients with initial CNS disease**

Patient	Age (years)	Gender	FAB	Leukocyte (/μL)	Cytogenetics	CNS Symptoms	Relapse	HSCT	Outcome
1	43	F	M1	1860	CN	Seizure	Yes^*#*^	Yes	CR in 52.7 m
2	21	F	M2	15430	t(8;21)	Blurred vision	No	Yes	CR in 41.4 m
3	60	F	M4	74390	t(9;11)(p22;q23)	Dizziness	-^*∆*^	No	Died in 3 m
4	26	F	M4	175900	inv(16)	Headache	No	No	CR in 63.1 m
5	72	M	M4	168630	inv(16)	Dizziness	No	No	CR in 40.9 m
6	53	F	M4	93340	+8	Paresthesias	No	No	Died in 4.3 m
7	40	M	M1	277250	CN	Headache	No	No	CR in 58.1 m

^*#*^This patient suffered from marrow relapse but not CNS relapse.

^*∆*^This patient suffered from induction death after standard induction chemotherapy.

Abbreviation: F, female; M, male; FAB, French-American-British; CN, normal karyotype; CNS, central nervous system; HSCT, hematopoietic, stem cell transplantation; CR, complete remission; m, month.

**Figure 1:**
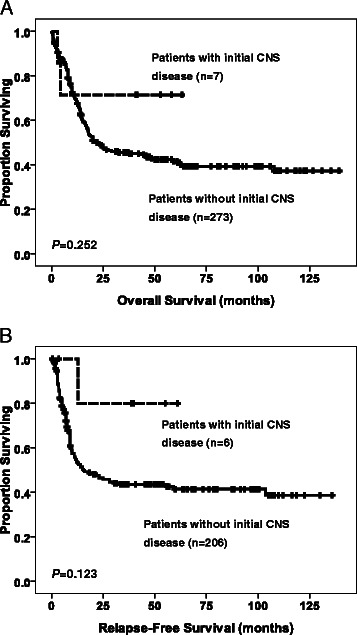
Kaplan-Meier curves for overall survival **(A)** and relapse-free survival **(B)** of AML patients ≥ 18 years of age stratified based their status of CNS involvement at diagnosis. Only those receiving conventional standard chemotherapy were included in the survival analyses.

The CR rates and primary refractory rates amongst patients with initial CNS disease were similar to those of patients without initial CNS disease (Additional file 1: Table S3). The median follow-up interval was 58.1 months (range: 0.1-139.3). The 5-year OS and RFS rates of the patients with initial CNS disease were not significantly different to those of the patients without initial CNS disease (*P* = .252 and *P* = .123, respectively, Figure 1).

### Clinical characteristics and outcomes of patients with isolated CNS relapse

**Figure 2:**
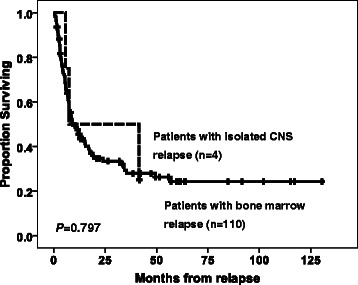
Kaplan-Meier curves for overall survival of AML patients ≥ 18 years of age with an isolated CNS relapse or a bone marrow relapse.

The clinical characteristics and treatment outcomes of the four AML patients who suffered an isolated CNS relapse are listed in Table 5. None of these patients had initial CNS disease. The median interval from attaining CR status to the first detection of isolated CNS relapse was 3.2 months. The patients who suffered an isolated CNS relapse were younger, had higher WBC counts and were more likely to have FAB-M4-subtype morphology than the patients without an isolated CNS relapse (*P* = .008, *P* = .022, and *P* = 0.044, respectively). The 11q23 abnormalities were more prevalent amongst patients who suffered an isolated CNS relapse than in patients without CNS relapse (*P* < .001). Three of four patients who suffered an isolated CNS relapse developed a subsequent BM relapse and died. The OS rate following an isolated CNS relapse was as poor as that following a BM relapse, with a median interval from isolated CNS relapse to death of 8.5 months (Figure 2).

**Table 5:** **Clinical characteristics and treatment outcomes of patients with isolated CNS relapse**

Patient	Age (years)	Gender	FAB	Leukocyte (/μL)	Cytogenetics	Initial CNS disease	CNS Symptoms	Radiation therapy*	HSCT	Time from remission to CNS relapse (months)	Time from CNS relapse to BM relapse (months)	Outcome after isolated CNS relapse
1	24	M	M5	113510	t(9;11;13)(p22;q23;q34)	Nil	Headache	No	Yes	2	-	CR in 41.6 m
2	28	M	M4	172700	t(11;19)(q23;p13)	Nil	Tremor	Yes	No	14	5.8	Died in 7.4 m
3	28	F	M4	123550	t(6;11)(q27;q23)	Nil	Dizziness	No	Yes	3.3	14.9^*#*^	Died in 41.4 m
4	35	M	M4	33510	CN	Nil	Blurred vision	Yes	Yes	3.2	2.3	Died in 5.8 m

*All patients received intensive systemic chemotherapy and intrathecal chemotherapy.

^*#*^Bone marrow relapses of this patient happened after hematopoietic stem cell transplantation.

Abbreviation: *F* female, *M* male, *FAB* French-American-British, *CN* normal karyotype, *CNS* central nervous system, *HSCT* hematopoietic stem cell transplantation, *BM* bone marrow, *CR* complete remission, *m* month.

## Discussion

Our current study examined the rate of CNS involvement in a large cohort of adult AML patients. The incidence of CNS involvement amongst adult AML patients at our institution was 5.1%. This result is similar to that reported in other studies, that the incidence of CNS involvement concurrent with or subsequent to AML diagnosis in adults is uncommon [1,15,36]. The AML patients with CNS involvement were younger, exhibited higher WBC, platelet, and peripheral blood blast cell counts, and had FAB M4 morphology, and 11q23 abnormalities more frequently than did the AML patients without CNS involvement. By multivariate analysis, the risk factors significantly associated with CNS involvement included age ≤ 45 years, WBC counts ≥ 50,000/μL, and the presence of 11q23 abnormalities. No significant difference in 5-year OS and RFS rates was observed between the AML patients with initial CNS disease and those without it. In contrast, the prognosis of patients who suffered an isolated CNS relapse was as poor as that of patients who suffered a bone marrow relapse.

Young age has previously been considered as a risk factor for CNS involvement and it was also observed in this study [14,15,37]. Since certain FAB subtypes, such as M4 AML, or cytogenetic abnormalities, such as the chromosome 16 inversion, are commoner in younger adults and are also more frequently associated with extramedullary infiltrates, this is likely to at least partly explain the association between CNS disease and younger age found in this and other studies. A large leukaemic cell burden, as represented by high WBC counts and high serum LDH levels, has been shown to be significantly associated with CNS involvement [3,8,13-16]. We observed that AML patients with high WBC counts at diagnosis had a significantly higher incidence of CNS involvement (Table 3). However, the association between serum LDH levels and CNS involvement was not statistically significant in our study (*P* = 0.069).

Chromosomal changes or molecular gene mutations in AML have clinical implications. Karyotype abnormalities, such as the chromosome 16 inversion, chromosome 11q23 abnormality, trisomy 8, t(9;11) translocation, and hyperdiploidism have been shown to be significantly associated with CNS involvement in paediatric AML [7,8,10,14]. We observed that the chromosome 16 inversion was more prevalent amongst patients with initial CNS disease, whereas the 11q23 abnormalities were more common amongst patients that suffered an isolated CNS relapse. These findings are consistent with those of previous studies of paediatric AML patients [8,10]. On the other hand, no relevant molecular gene mutation associated with CNS involvement was identified in this study.

Reports of outcome about CNS involvement in adults with AML are limited. Chang et al reported that extramedullary infiltrates were associated with poor outcome in adult patients with AML, but their investigation did not focus specifically on CNS involvement [38]. Mayadev et al [39] showed that CNS involvement was associated with poor prognosis in adult AML patients. On the contrary, other two studies [16,37] showed that the outcomes for AML with CNS involvement were comparable with those for AML without CNS involvement. However, participant selection for these three studies was limited to patients who had undergone HSCT, which may have confounded their results. In our study, the 5-year OS and RFS of patients with initial CNS disease were similar to those of AML patients without CNS disease. These findings may be partially explained by the higher frequency of favorable cytogenetics (inversion of chromosome 16), the absence of CNS relapse, and low rate of BM relapse (16.7%, Table 4) amongst the patients with initial CNS disease. By contrast, our data demonstrated that the outcome of patients who suffered an isolated CNS relapse was actually poor. Three of four patients (75%) who suffered an isolated CNS relapse developed a subsequent BM relapse and died, despite receiving cranial irradiation or allogeneic HSCT.

No consensus exists regarding the treatment of AML patients with CNS involvement. The preference of treatment protocols used in our study is largely based on the capacity of intrathecal chemotherapy to clear the leukaemic cells of CSF quickly in most patients and the efficacy of high doses of cytarabine for penetrating the CNS [40,41]. Moreover, the potential acute and long-term complications associated with cranial irradiation often limit its use. Aoki et al [37] reported that allogeneic HSCT may improve outcomes for CNS involvement in patients with AML. However, further prospective studies are necessary to clarify this point. Future investigations of more effective CNS-directed treatment strategies are warranted to improve the outcomes of such patients, particularly those who suffer an isolated CNS relapse.

The limitation of our study is that this is a single centre, retrospective study. Nevertheless, most published studies concerning this subject have also been retrospective. Identifying adult patients with AML who are at risk for CNS involvement will enable us to restrict the use of CNS prophylactic therapy to those who are most likely to benefit. In this study three significant risk factors for CNS involvement in adult patients with AML were recognized. Further studies with large cohorts are necessary to validate this point.

## Conclusions

Our study of a large cohort of adult AML patients revealed that the incidence of CNS involvement is low. Age ≤ 45 years, WBC counts ≥ 50,000/μL and the presence of 11q23 abnormalities are independent risk factors for adult AML patients with CNS involvement either at diagnosis or during the course of the disease. The OS rate is similar between patients with initial CNS disease and those without it. However, the prognosis of patients who suffer an isolated CNS relapse is as poor as that of patients who suffered a bone marrow relapse. Whether routine CNS prophylactic therapy should be given as part of conventional standard chemotherapy in adult AML patients having these specific risk factors needs to be further investigated.

## Additional file

Additional file 1: Table S1. Comparison of immunophenotype of leukemia cells seen in patients with and without central nervous system involvement. **Table S2.** Comparison of molecular gene mutation* seen in patients with and without central nervous system involvement. **Table S3.** Comparison of treatment response of patients with and without initial CNS disease by standard remission induction therapy.

## Abbreviations

CNS: Central nervous system
AML: Acute myeloid leukaemia
WBC: White blood cell
PBC: Peripheral blast cell
FAB: French-American-British
NTUH: National Taiwan University Hospital
HSCT: Haematopoietic stem cell transplantation
CSF: Cerebrospinal fluid
CR: Complete remission
BM: Bone marrow
LDH: Lactate dehydrogenase
OR: Odds ratio
OS: Overall survival
RFS: Relapse-free survival
