# Supplementary material for: Risk factors and clinical outcomes of acute myeloid leukaemia with central nervous system involvement in adults
Source: BMC Cancer. 2015 May 2;15:344. doi: 10.1186/s12885-015-1376-9 (PMC4419415; doi:10.1186/s12885-015-1376-9)
Supplement: Additional file 1: Table S1. — Comparison of immunophenotype of leukemia cells seen in patients with and without central nervous system involvement. Table S2. Comparison of molecular gene mutation* seen in patients with and without central nervous system involvement. Table S3. Comparison of treatment response of patients with and without initial CNS disease by standard remission induction therapy. [file 12885_2015_1376_MOESM1_ESM.doc]

**Supplementary Table 1 Comparison of immunophenotype of leukemia cells seen in patients with and without central nervous system involvement**

| **Antigens** | **Percentage of patients with the**  **antigen expression** | | | | ***P* value** |
| --- | --- | --- | --- | --- | --- |
|  | **Whole cohort** | **Patients with**  **CNS involvement** | | **Patients without**  **CNS involvement** |  |
| HLA-DR | 74.7(287/384) | 68.4(13/19) | 75.1(274/365) | | 0.588 |
| CD13 | 94.8(364/384) | 84.2(16/19) | 95.3(348/365) | | 0.069 |
| CD33 | 91.4(351/384) | 94.7(18/19) | 91.2(333/365) | | >0.999 |
| CD34 | 71.5(273/382) | 78.9(15/19) | 71.1(258/363) | | 0.606 |
| CD11b | 33.3(32/96) | 0(0/3) | 34.4(32/93) | | 0.548 |
| CD14 | 12.1(45/371) | 0(0/17) | 12.7(45/354) | | 0.243 |
| CD19 | 5.8(22/377) | 5.3(1/19) | 5.9(21/358) | | >0.999 |
| CD7 | 20.7(79/381) | 16.7(3/18) | 20.9(76/363) | | >0.999 |
| CD2 | 1.6(6/380) | 5.6(1/18) | 1.4(5/362) | | 0.254 |
| CD15 | 39.4(150/381) | 44.4(8/18) | 39.1(142/363) | | 0.632 |
| CD56 | 23.3(88/377) | 21.1(4/19) | 23.5(84/358) | | >0.999 |

Abbreviation: CNS, central nervous system.

**Supplementary Table 2 Comparison of molecular gene mutation^*^ seen in patients with and without central nervous system involvement**

| **Variables** | **Percentage of patients with the gene mutation** | | | ***P* value** |
| --- | --- | --- | --- | --- |
|  | **Whole cohort** | **Patients with**  **CNS involvement** | **Patients without**  **CNS involvement** |  |
| *CEBPA* | 14.4(56/390) | 5.9(1/17) | 14.7(55/373) | 0.486 |
| *FLT3-*ITD | 21.3(83/390) | 29.4(5/17) | 20.9(78/373) | 0.375 |
| *NPM1* | 20.3(79/390) | 11.8(2/17) | 20.6(77/373) | 0.542 |
| *N-RAS* | 12.1(47/387) | 23.5(4/17) | 11.6(43/370) | 0.138 |
| *K-RAS* | 3.4(13/387) | 0(0/17) | 3.5(13/370) | >0.999 |
| *MLL-*PTD | 6.3(24/383) | 0(0/17) | 6.6(24/366) | 0.614 |
| *KIT* | 3.3(12/369) | 0(0/16) | 3.4(12/353) | >0.999 |
| *WT1* | 6.0(22/369) | 6.3(1/16) | 5.9(21/353) | >0.999 |
| *AML1/RUNX1* | 12.4(48/388) | 5.9(1/17) | 12.7(47/371) | 0.707 |
| *ASXL1* | 12.2(47/385) | 6.3(1/16) | 12.5(46/369) | 0.705 |
| *IDH1* | 6.2(24/386) | 6.3(1/16) | 6.2(23/370) | >0.999 |
| *IDH2* | 12.5(48/385) | 6.3(1/16) | 12.7(47/369) | 0.705 |
| *TET2* | 12.5(46/368) | 13.3(2/15) | 12.5(44/353) | >0.999 |
| *DNMT3A* | 17.2(63/366) | 13.3(2/15) | 17.4(61/351) | >0.999 |

**^*^**Because of missing data, we do not have complete information of molecular gene mutations of every patient in our cohort.

Abbreviation: ITD, internal tandem duplication; PTD, partial tandem duplication.

**Supplementary Table 3 Comparison of treatment response of patients with and without initial CNS disease by standard remission induction therapy**

| **Variables** | **Total** | **Patients with initial CNS disease** | **Patients without initial CNS disease** | ***P* value** |
| --- | --- | --- | --- | --- |
| **Induction response**†*^∆^* | 280 | 7 | 273 |  |
| CR | 202 | 6(85.7) | 196(71.8) | 0.677 |
| Refractory | 60 | 0(0) | 60(22) | 0.352 |
| Induction death | 18 | 1(14.3) | 17(6.2) | 0.375 |

† number of patients (%)

*^∆^*Only the 280 patients who received conventional intensive induction chemotherapy and then

consolidation chemotherapy if CR was achieved, were included in the analysis.

Abbreviation: CNS, central nervous system; CR, complete remission.
